# Supplementary material for: Tuning the effective spin-orbit coupling in molecular semiconductors
Source: Nat Commun. 2017 May 11;8:15200. doi: 10.1038/ncomms15200 (PMC5437270; doi:10.1038/ncomms15200)
Supplement: Supplementary Information — Supplementary Figures, Supplementary Tables, Supplementary Notes and Supplementary References [file ncomms15200-s1.pdf]

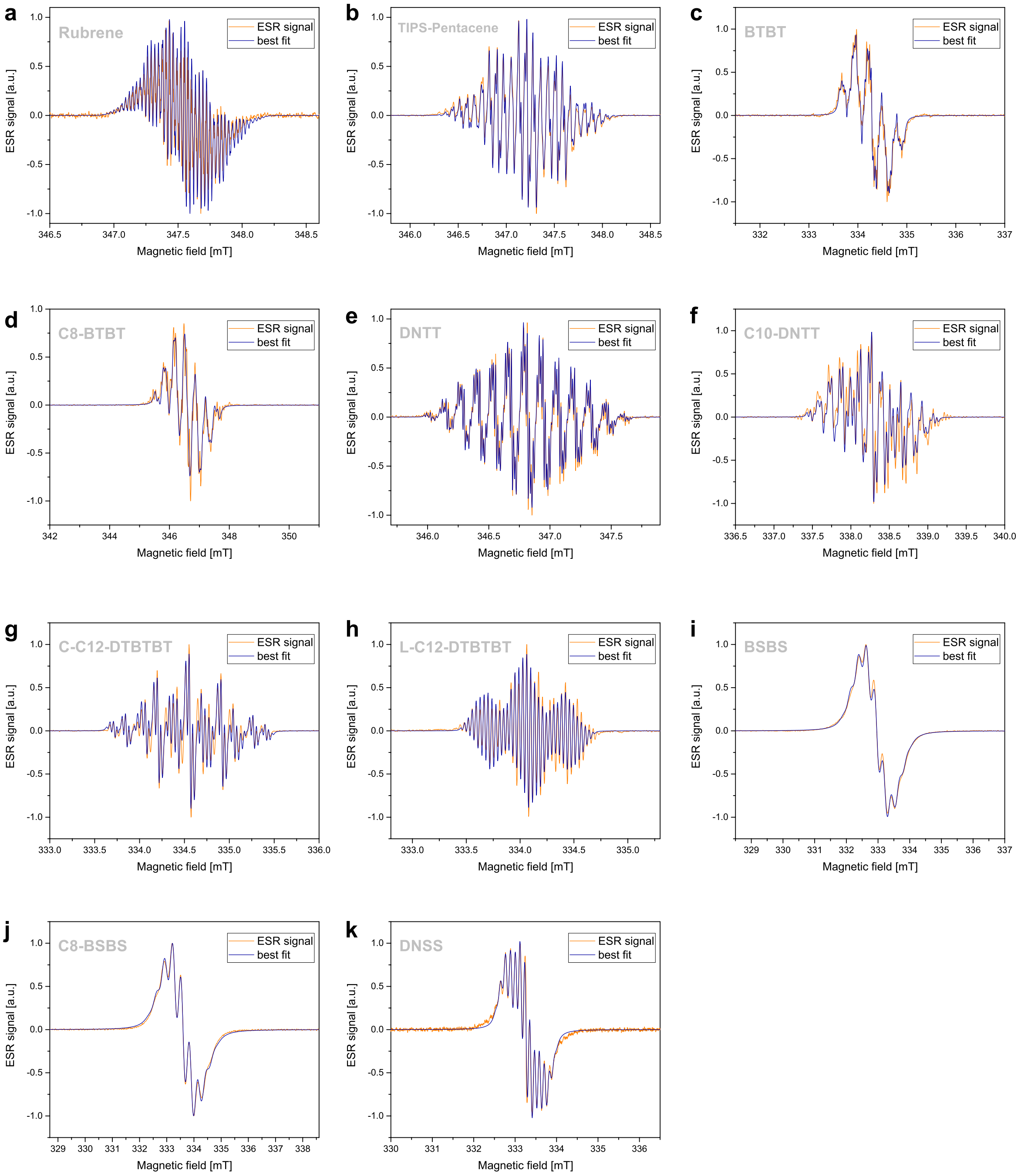

Supplementary Figure 1: Overview of all recorded ESR spectra. Microwave powers were chosen sufficiently small to prevent any saturation of the signal (typically 0.006–$0.06\,\mathrm{mW}$) and the modulation amplitude was chosen smaller than the line width to prevent distortions of the spectrum.

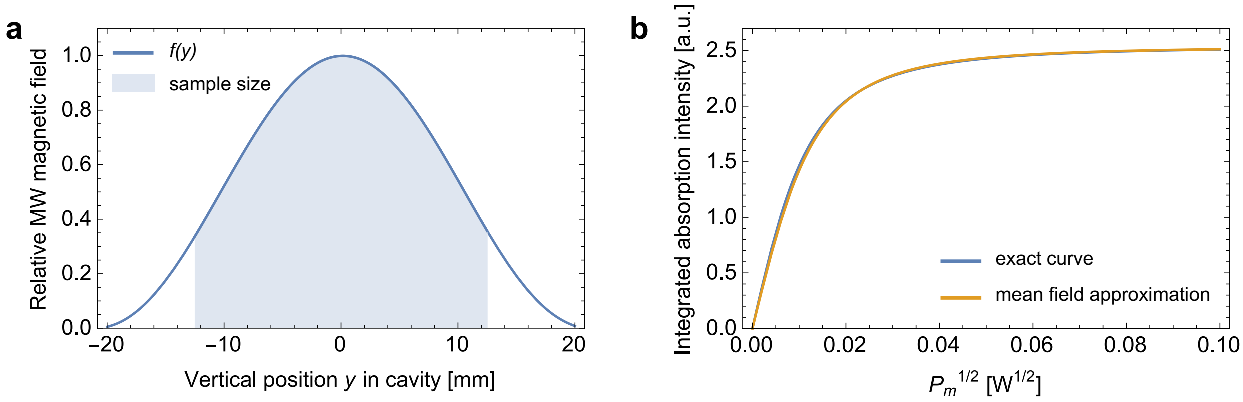

Supplementary Figure 2: Microwave field distribution in cavity. (**a**) Microwave magnetic field distribution $f(y)$ inside the Bruker ER 4122SHQE cavity. The shaded area is representative of a 25 mm long sample. (**b**) Exact power saturation curve from Eq. 6 taking in to account the variation in signal intensity for different positions in the cavity and power saturation behavior of a sample that only sees a uniform average microwave field $\overline{B_1(y)}$. Shown curves are for $L = 25$ mm since any visible difference disappears for smaller L.

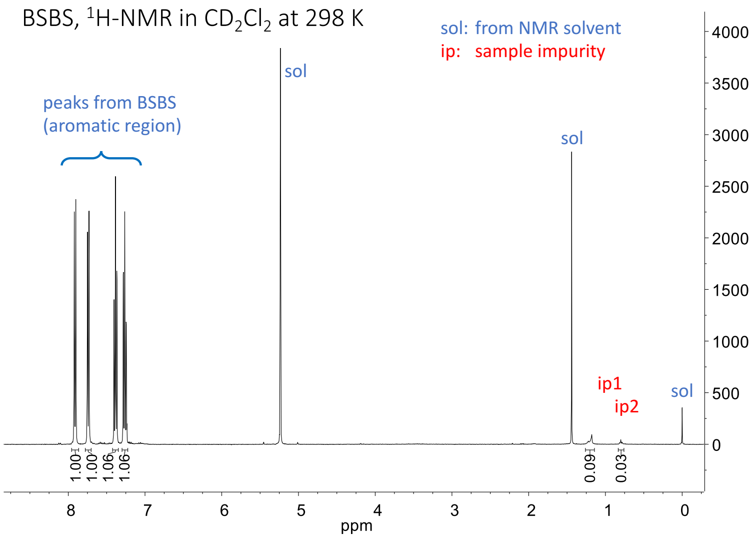

Supplementary Figure 3: NMR spectrum composition for BSBS. ^{1}H-NMR spectrum of BSBS dissolved in BSBS in deuterated dichloromethane (CD_2Cl_2) at a concentration of $0.02\,\mathrm{mg\,mL^{-1}}$ with small impurity peaks in the aliphatic region labeled ip1 and ip2. The spectrum was recorded on a Bruker AV-400 spectrometer at 298 K in deuterated dichloromethane and is reported in ppm relative to tetramethylsilane (TMS).

Acronym	Chemical name	Structure	Δg [ppm]	$\Delta g^{OZ/SOC}$ [ppm]
pentacene	pentacene	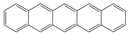	352	346
rubrene	rubrene	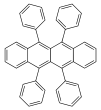	81	73
BTBT	[1]Benzothieno[3,2-b][1]benzothiophene	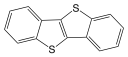	2238	2216
C8-BTBT	2,7-Dioctyl[1]benzothieno[3,2-b][1]benzothiophene	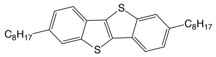	1107	1109
C8*s*-BTBT	3,8-Dioctyl[1]benzothieno[3,2-b][1]benzothiophene	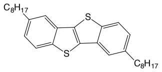	2828	2812
BSBS	[1]Benzoseleno[3,2-b][1]benzoselenophene	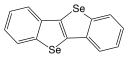	14255	14129
C8-BSBS	2,7-Dioctyl[1]benzoseleno[3,2-b][1]benzoselenophene	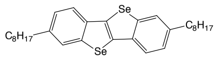	6773	6696
C8*s*-BSBS	3,8-Dioctyl[1]benzoseleno[3,2-b][1]benzoselenophene	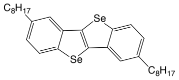	16677	16548
DNTT	Dinaphtho[2,3-b:2’,3’-f]thieno[3,2-b]thiophene	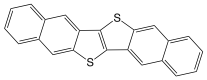	2073	2035
C8-DNTT	2,9-Dioctyldinaphtho[2,3-b:2’,3’-f]thieno[3,2-b]thiophene	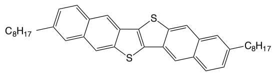	1794	1778
C8*s*-DNTT	3,10-Dioctyldinaphtho[2,3-b:2’,3’-f]thieno[3,2-b]thiophene	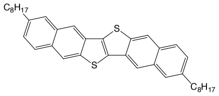	1769	1754
DNSS	Dinaphtho[2,3-b:2’,3’-f]seleno[3,2-b]selenophene	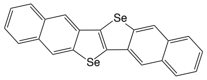	10414	10289
C8-DNSS	2,9-Dioctyldinaphtho[2,3-b:2’,3’-f]seleno[3,2-b]selenophene	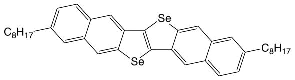	8760	8662
C8*s*-DNSS	3,10-Dioctyldinaphtho[2,3-b:2’,3’-f]seleno[3,2-b]selenophene	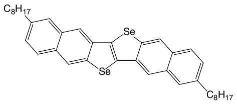	8526	8429
DATT	Dianthra[2,3-b:2’,3’-f]thieno[3,2-b]thiophene	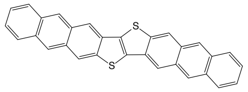	1598	1553
C8-DATT	2,11-Dioctyldianthra[2,3-b:2’,3’-f]thieno[3,2-b]thiophene	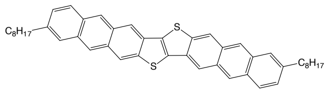	1481	1459
C8*s*-DATT	3,12-Dioctyldianthra[2,3-b:2’,3’-f]seleno[3,2-b]selenophene	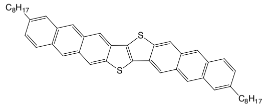	1527	1506

Supplementary Table 1: Molecular g-tensor shifts Δg calculated with DFT, and the corresponding $\Delta g^{OZ/SOC}$ terms (see Eq. 2).

Acronym	Chemical name	Structure	Δg [ppm]	$\Delta g^{OZ/SOC}$ [ppm]
DASS	Dianthra[2,3-b:2',3'-f]seleno[3,2-b]selenophene	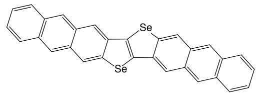	7031	6911
C8-DASS	2,11-Dioctyldianthra[2,3-b:2',3'-f]seleno[3,2-b]selenophene	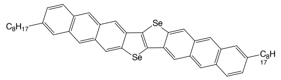	6415	6321
C8*s*-DASS	3,12-Dioctyldianthra[2,3-b:2',3'-f]seleno[3,2-b]selenophene	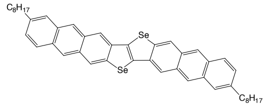	6620	6526
C-DTBTBT	Dibenzothiopheno[7,6-b:7',6'-f]thieno[3,2-b]thiophene	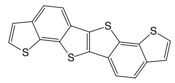	419	383
C-C8-DTBTBT	2,8-Dioctyldibenzothiopheno[7,6-b:7',6'-f]thieno[3,2-b]thiophene	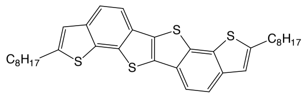	237	231
C-C8*s*-DTBTBT	3,9-Dioctyldibenzothiopheno[7,6-b:7',6'-f]thieno[3,2-b]thiophene	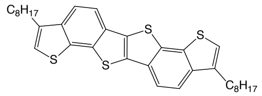	802	759
C-DSBSBS	Dibenzoselenopheno[7,6-b:7',6'-f]seleno[3,2-b]selenophene	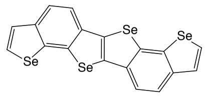	2498	2395
C-C8-DSBSBS	2,8-Dioctyldibenzoselenopheno[7,6-b:7',6'-f]seleno[3,2-b]selenophene	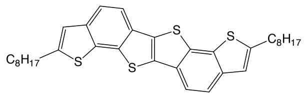	1117	1041
C-C8*s*-DSBSBS	3,9-Dioctyldibenzoselenopheno[7,6-b:7',6'-f]seleno[3,2-b]selenophene	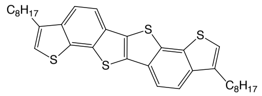	4672	4547
L-DTBTBT	Dibenzothiopheno[6,5-b:6',5'-f]thieno[3,2-b]thiophene	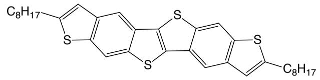	4176	4128
L-C8-DTBTBT	2,8-Dioctyldibenzothiopheno[6,5-b:6',5'-f]thieno[3,2-b]thiophene	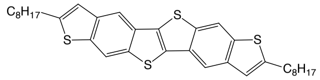	4180	4143
L-C8*s*-DTBTBT	3,9-Dioctyldibenzothiopheno[6,5-b:6',5'-f]thieno[3,2-b]thiophene	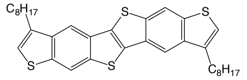	4166	4105
L-DSBSBS	Dibenzoselenopheno[6,5-b:6',5'-f]seleno[3,2-b]selenophene	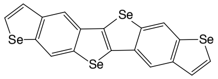	23265	23082
L-C8-DSBSBS	2,8-Dioctyldibenzoselenopheno[6,5-b:6',5'-f]seleno[3,2-b]selenophene	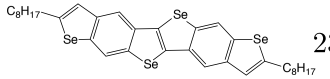	23527	23353
L-C8*s*-DSBSBS	3,9-Dioctyldibenzoselenopheno[6,5-b:6',5'-f]seleno[3,2-b]selenophene	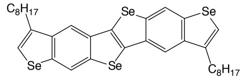	23082	22882

Supplementary Table 1: Molecular g-tensor shifts Δg calculated with DFT, and the corresponding $\Delta g^{OZ/SOC}$ terms (see Eq. 2).

Element	c_e avg.	c_e std. dev.
C	$1.31 \cdot 10^{-05}$	$9.08 \cdot 10^{-06}$
S	$9.10 \cdot 10^{-03}$	$6.72 \cdot 10^{-05}$
Se	$3.47 \cdot 10^{-02}$	$1.32 \cdot 10^{-04}$

Supplementary Table 2: Average and standard deviations of the dimensionless proportionality constants c_e fitted for the $\Delta g^{OZ/SOC}$ terms of the set of molecules presented in Supplementary Table 1.

Supplementary Note 1: Theoretical DFT calculations

This section provides additional detail on the theoretical calculations performed. As stated in the main text, the theoretical work is intended to support the key message that *g*-shifts in the studied class of molecules may be understood in terms of the effective spin-orbit coupling, or equivalently, the degree of overlap between the orbital- and spin angular momentum distributions.

We argue that, given simple geometries and / or weaker SOC, we may predict the dominant $\Delta g^{OZ/SOC}$ term of the *g*-tensor shifts from a linear model of atomically localized SOC interactions. The latter amounts to a linear fit of atomically localized spin populations to the $\Delta g^{OZ/SOC}$ terms, on the form of Eq. 3. In other words, if a linear model with the distribution of atomic spin population as only independent variable is predictive, we consider our hypothesis confirmed. We validate the model by studying the correlation between the fitted $\Delta g^{OZ/SOC}$ values, and those calculated from first-principles.

The theoretical methodology follows three steps, i) DFT calculations of molecular geometries, cation spin densities, and *g*-tensors, ii) calculations of atomic spin populations using an atom-in-molecule (AIM) decomposition method, and iii) a multivariate linear regression of the calculated dominant $\Delta g^{OZ/SOC}$ *g*-shift term versus atomic spin populations. These three steps are described in reproducible detail in the following paragraphs.

DFT Calculations

We use DFT to predict *g*-tensor shifts from first-principles theory. These shifts depend on spin-orbit coupling (SOC), ranging from weak and nearly insignificant in the pure hydrocarbons studied, to significant in the Se-substituted molecules. We therefore require an accurate and transferable level of theory, able to describe a wide range of SOC strengths on a consistent footing.

Consequentially, we opted for an all-electron DFT method, with nuclear relativistic effects described by the zeroth-order regular approximation (ZORA[1]) using the standard point-charge approximation for the atomic nuclei. *g*-tensors were calculated using the method[2] developed by Neese et al. and related techniques[3] as implemented in the ORCA software package[4], version 3.0.3. Tests showed hybrid exchange-correlation functionals in general, and the PBE0[5] parameterization in particular, to perform very well for the prediction of the experimentally measured *g*-tensor shifts, in agreement with previous findings[2]. Exhaustive tests of the SARC basis set family[6] revealed that no qualitative features improved past the triple-zeta level (a.k.a. the 'ma-zora-def2-tzvp' basis set). The benchmark level quadruple-zeta 'ma-zora-def2-qzvp' basis set gives slightly larger (on the order of hundreds of [ppm]) *g*-shifts. Therefore, while the 'ma-zora-def2-tzvp' basis set, with linear dependencies eliminated by removing the diffuse (a.k.a. 'minimal augmentation') functions on the carbon atoms represents the optimal balance between accuracy of predictions and computational cost, the accuracy of predicted *g*-shifts is partially due to error cancellation between the basis set and other, over-estimating factors.

All molecules were fully geometry optimized in a charge neutral state and the above described level of theory including all-electron spin-orbit coupling, using the NWChem quantum chemistry software package[7], version 6.5. Compared to a non-relativistic, spin-orbit free level of theory, the influence of the ZORA on the geometry is significant, but SOC effects negligible. Geometry differences between molecules optimized in a neutral and charged state were small, allowing for greater comparability by obtaining all geometries in the neutral state. The optimized pentacene and rubrene geometries have D_{2H} point-group symmetry. All other optimized molecules belong to the C_{2H} point-group. While experimental measurements were performed on molecules of varying alkyl chain

lengths, e. g. C10, C12 etc., all molecules in the theoretical calculations were given 8-membered alkyl chains. Tests showed the alkyl chain influence to be well converged at this length, meaning that the theoretical results presented here are comparable to experimental measurements at chains equal to or longer than 8 methylene units.

For each optimized geometry, a single-point calculation with a total charge of +1 and multiplicity 2 was performed, with a subsequent calculation of the g-tensor and dumping of the DFT spin density onto a dense regular grid. The resulting calculated g-tensor shifts are shown in Supplementary Table 1 in parts per million (ppm). Evidently, the total g-tensor shift $\Delta \mathrm{g}$ is strongly dominated by the orbital Zeeman / SOC-dependent term $\Delta \mathrm{g}^{\mathrm{OZ/SOC}}$ in the studied class of molecules. That is, as mentioned in the main text, the gauge correction and relativistic mass correction terms add up to a very small, mostly positive term (67 ppm on average in Supplementary Table 1).

Atomic Spin Calculations

In the main text we show, that g-tensor shifts in the studied class of molecules can be understood in terms of a linear relationship between the dominant $\Delta \mathrm{g}^{\mathrm{OZ/SOC}}$ term, and the spin density distribution among the nuclei in the molecule. More specifically, $\Delta \mathrm{g}^{\mathrm{OZ/SOC}}$ is approximated as a sum over fitted proportionality constants representing the effective orbital angular momentum coupling for a given element, multiplied with sums of atomic spin populations at that element. This requires calculating atomic spin populations from the molecular spin density, in other words, finding an appropriate solution to the atoms-in-molecules (AIM) problem.

The AIM problem is commonly solved using methods partitioning either a) wave-functions based on projections of basis functions, or b) charge- or spin-densities based on their spatial distribution. The first option, exemplified by the Mulliken and Löwdin[8] population analysis methods, are conceptually simpler and readily implemented, but suffer from significant basis set effects[9,10]. Tests using the arguably more rigorous Löwdin method confirmed significant variations in calculated atomic spin populations with basis set type and size. The basis set dependence also manifested in a sensitive dependence on relative nuclear coordinates, appearing as an unexplained scatter in correlation plots of data fitted according to the procedure below. The second option, exemplified by the Bader method[11], does not depend on the basis set used in the calculation of the molecular spin density. However, for the current set of molecules, the Bader method did not prove significantly more stable with respect to small variations in molecular structure than the above mentioned Löwdin method, nor did using the alternative weighting method proposed by Yu and Trinkle[12] alleviate the problem.

The Voronoi Deformation Density (VDD) method[10] proved significantly more stable and consistent, however, and is the method used in all fits of $\Delta \mathrm{g}^{\mathrm{OZ/SOC}}$ presented in this manuscript. All density-based partitioning methods were applied using the 'bader' program developed by Henkelman et al.[13], version 0.95a.

Fit of $\Delta \mathrm{g}^{\mathrm{OZ/SOC}}$ vs Atomic Spins

For completeness, the fitted c_e are presented in Supplementary Table 2. Since each molecule is excluded from the fitting set when fitted for, the c_e are in principle unique for each molecule. Consequentially, Supplementary Table 2 shows the average c_e for each element in the complete set of 32 molecules studied, and the corresponding standard deviation.

As expected of a parameter representing the net effect of orbital angular momentum, c_e increases dramatically going from carbon via sulphur to selenium. The reasons for the precise magnitude

of the c_e are convoluted, and we will here refrain from a detailed interpretation. It is however noteworthy that the fit is relatively consistent for the heavier elements, with standard deviations below a percent of the average value. Because of it's very small contributions to the $\Delta g^{\mathrm{OZ/SOC}}$ combined with the limited precision of input data, the carbon fits are considerably less numerically consistent, resulting in a relative standard deviation of approximately 70 percent.

Supplementary Note 2: Deviation of g-shifts for BSBS

All g-factor factors show an excellent agreement between experiment and theory apart from the values determined for BSBS. A systematic error in the DFT calculations is unlikely since only BSBS shows a large discrepancy. We consequently tried to eliminate all error sources for the ESR measurements. To rule out aggregation of the molecules in solution, we reduced the concentration of BSBS close to the detection limit of the instrument ($0.05 \times 10^{-3}\,\mathrm{mol\,L^{-1}}$ or 4×10^{14} spins in the sample tube) and heated up the solution to $38\,^{\circ}\mathrm{C}$ (boiling point of DCM: $39.6\,^{\circ}\mathrm{C}$) during the measurement. Neither resulted in any changes of the g-factor. In a control experiment, we investigated the solutions at different concentrations using diffusion-ordered nuclear magnetic resonance spectroscopy (DOSY NMR). These spectra show that the diffusion constant of BSBS increases marginally from $1.8 \times 10^{-9}\,\mathrm{m^2\,s^{-1}}$ to $2.1 \times 10^{-9}\,\mathrm{m^2\,s^{-1}}$ when reducing its concentration from $28 \times 10^{-3}\,\mathrm{mol\,L^{-1}}$ to $0.28 \times 10^{-3}\,\mathrm{mol\,L^{-1}}$. This indicates the absence of aggregation which should otherwise result in a strong decrease of diffusion constants with increasing concentrations.

Consistent with the difference in g-factors, we also observe different HFI coupling constants in theory and experiment: BSBS shows a root mean square (rms) deviation of ~ 3 between theoretical and experimental HFI couplings compared to rms deviations < 0.8 for all other molecules. Aggregation seems unlikely as the cause for this difference, especially since the spread of the spin density over more than one molecule would increase the number of protons participating in the HFI. At present, we are not able to pinpoint the source of the discrepancy, apart from possible defects in the molecular structure. The hydrogen NMR spectrum (Supplementary Fig. 3) reveals small impurity peaks in the aliphatic region though it remains unclear at present if they could cause such a strong shift in spin density.

Supplementary Note 3: ESR power saturation measurements

When the decay of spin polarization is an exponential process, it is possible to define spin lifetimes T_1 and T_2 and describe the time evolution of the spins by Bloch equations. The absorption rate of microwaves then follows a Lorentzian profile[14]

$$P(\omega) = \frac{n_0}{2} \frac{\hbar\omega\gamma_e^2 B_1^2 T_2}{1 + T_2^2(\omega_0 - \omega)^2 + \gamma_e^2 T_1 T_2 B_1^2} \tag{1}$$

where n_0 is the population difference between Zeeman split levels, $2\pi\hbar$ is the Planck constant, γ_e the gyromagnetic ratio of an electron, ω the microwave frequency, B_1 the microwave magnetic field and $\omega_0 = \gamma_e B_0$ the Larmor frequency in an external magnetic field B_0. $P(\omega)$ scales with B_1^2 in the absence of power saturation. The ESR signal is detected by a Schottky diode crystal and the detector current varies with the square root of the microwave power, i.e., linearly with B_1:

$$\text{ESR Intensity}\,(B_0) \propto \chi_0 \frac{\omega B_1 T_2}{1 + T_2^2(\gamma_e B_0 - \omega)^2 + \gamma_e^2 T_1 T_2 B_1^2} \tag{2}$$

Here, χ_0 is the paramagnetic susceptibility of the sample. All measurements are recorded by a lock-in amplifier with a modulated external field B_0 and are thus recorded as a derivative of Eq. 2 with respect to B_0.

It is possible to determine the product T_1T_2 of the two lifetimes by saturating the ESR signal with increasing microwave powers. The integral of Eq. 2 over all fields B_0, or the double integral over the recorded derivative spectrum, scales with the microwave magnetic field B_1 as[15,16]

$$\text{DI}(B_1) \propto \chi_0 \frac{B_1}{\sqrt{1 + \gamma_e^2 T_1 T_2 B_1^2}} \ . \tag{3}$$

The measured power saturation curves together with the fits to Eq. 3 are shown Fig. 3b.

Before the onset of power saturation and line width broadening, the coherence time is given by $T_2 = (\gamma_e \Delta B_{1/2})^{-1}$ where $\Delta B_{1/2}$ is the half width at half maximum of the Lorentzian resonance[15]. In practice, we use the line widths of the individual Lorentzian resonances that make up the spectrum, determined by the least-squares fitting of the hyperfine structure. Dividing the product of spin lifetimes by T_2 then gives the spin lattice relaxation time T_1.

Microwave magnetic field in the cavity:

Determining the product T_1T_2 relies on the knowledge of B_1 over the sample volume while only the applied power P_{mw} is recorded during an experiment. The scaling of B_1 with $\sqrt{P_{\text{mw}}}$ depends on the resonator architecture and losses inside the cavity.

In the absence of power saturation, the integrated absorption signal of a paramagnetic sample scales only with its magnetic susceptibility and the field amplitude B_1 at the sample position. This allows us to determine B_m from the integrated absorption signal of a reference sample with a known susceptibility. For the center of the cavity, we find as a function of the cavity Q-value and the incident microwave power P_m:

$$B_1^{\text{center}} = c\,\sqrt{QP_{\text{mw}}}\ , \quad c = 2.4069 \times 10^{-6}\,\text{T}\,\text{W}^{-1/2} \ . \tag{4}$$

The vertical microwave magnetic field distribution $f(y)$ is given by Bruker as a 9th order polynomial and stored with each data acquisition file (Supplementary Fig. 2a). We additionally confirmed the

values by monitoring the ESR signal intensity for a point like marker sample for different positions in the cavity (Fig. 3a). Since our samples are confined in a capillary tube with a radius of 0.5 mm around the cavity center, we only correct for the vertical field distribution by calculating the average microwave field across the sample length L and neglected any radial variations:

$$\overline{B_1(y)} = \frac{1}{L}\int_{-L/2}^{L/2} f(y) B_1^{\mathrm{center}}\; dy \tag{5}$$

A small error is introduced when using the average over the sample volume to fit the power saturation curves with Eq. 3 because we do not take into account that parts of the sample at positions with a lower field will contribute less to the total power saturation curve. In fact, the precise expression for the observed power saturation behavior would be

$$A(P_{\mathrm{mw}}) = \frac{1}{V}\int_{-L/2}^{L/2} \mathrm{DI}\left(f(y)B_1^{\mathrm{center}}\right)\cdot f(y)\,dy \text{ with } V = \int_{-L/2}^{L/2} f(y)\; dy\ . \tag{6}$$

as opposed to our approximation:

$$A(P_{\mathrm{mw}}) \approx \mathrm{DI}(\overline{B_1(y)}) = \mathrm{DI}(\overline{f(y)}c\sqrt{QP_{\mathrm{mw}}})\ . \tag{7}$$

However, the resulting error for our sample length of $L = 20$ mm remains well below 1% because the volume fraction at positions $y \neq 0$ is double that of the volume fraction at $y = 0$. A comparison of estimated and exact power saturation curves for a 25 mm long sample is shown in Supplementary Fig. 2b, any visible differences disappear completely for sample lengths $\sim$20 mm.

We also neglect the (small) distortion of the microwave field due to the sample's dielectric properties and therefore calculate with an error of $\pm 8\%$ in $T_1 T_2$ from the uncertainty in B_1 in addition to the standard error of from fitting.

Supplementary Note 4: DOSY NMR: diffusion constants and correlation times

DOSY NMR spectra were recorded on a Bruker AV-500 spectrometer at 298 K in deuterated dichloromethane. The diffusion experiments were performed using the Bruker ledbpgp2s pulse sequence. The number of data points per experiment was 32000 (td2) and the number of scans was 192. 24 experiments (td1) were collected using a diffusion delay (Δ) of 28 ms (d20) and $\delta/2$ pulse of 1.3 ms (p30).

The translational diffusion constants D_{tr} from DOSY NMR allow us to calculate the hydrodynamic volume and Stokes radius r_S of the respective molecule by the Stokes-Einstein equation

$$D_{\mathrm{tr}} = \frac{k_B T}{6\pi\eta r_S} \tag{8}$$

with the Boltzmann constant k_B, the sample temperature $T = 295\,\mathrm{K}$ and the viscosity of DCM $\eta = 4.13 \times 10^{-4}\,\mathrm{kg\,m^{-1}\,s^{-1}}$ at room temperature. Knowledge of the hydrodynamic volume then allows us to estimate the rotational diffusion constant

$$D_{\mathrm{rot}} = \frac{k_B T}{8\pi\eta r_S^3} \tag{9}$$

and the corresponding rotational correlation time, i.e., the time scale for which the rotational motion of the molecule is completely randomized, which is given by[14,15]:

$$\tau_{\mathrm{C}}^{\mathrm{diff}} = \frac{1}{6D_{\mathrm{rot}}} = \frac{2}{9}\left(\frac{k_B T}{\pi\eta}\right)^2 \frac{1}{D_{\mathrm{tr}}^3} \tag{10}$$

Supplementary References

1. van Lenthe, E., Baerends, E. J. & Snijders, J. G. Relativistic regular two-component Hamiltonians. *J. Chem. Phys.* **99**, 4597–4610 (1993).

2. Neese, F. Prediction of electron paramagnetic resonance g values using coupled perturbed Hartree–Fock and Kohn–Sham theory. *J. Chem. Phys.* **115**, 11080–11096 (2001).

3. Neese, F. Efficient and accurate approximations to the molecular spin-orbit coupling operator and their use in molecular g-tensor calculations. *J. Chem. Phys.* **122**, 034107 (2005).

4. Neese, F. The ORCA program system. *WIREs Comput. Mol. Sci.* **2**, 73–78 (2012).

5. Adamo, C. & Barone, V. Toward chemical accuracy in the computation of NMR shieldings: the PBE0 model. *Chem. Phys. Lett.* **298**, 113–119 (1998).

6. Pantazis, D. A., Chen, X.-Y., Landis, C. R. & Neese, F. All-Electron Scalar Relativistic Basis Sets for Third-Row Transition Metal Atoms. *J. Chem. Theory Comput.* **4**, 908–919 (2008).

7. Valiev, M. *et al.* NWChem: A comprehensive and scalable open-source solution for large scale molecular simulations. *Comput. Phys. Commun.* **181**, 1477–1489 (2010).

8. Löwdin, P.-O. On the Non-Orthogonality Problem Connected with the Use of Atomic Wave Functions in the Theory of Molecules and Crystals. *J. Chem. Phys.* **18**, 365–375 (1950).

9. Philips, J. J., Hudspeth, M. A., Browne, P. M. & Peralta, J. E. Basis set dependence of atomic spin populations. *Chem. Phys. Lett.* **495**, 146–150 (2010).

10. Fonseca Guerra, C., Handgraaf, J.-W., Baerends, E. J. & Bickelhaupt, F. M. Voronoi deformation density (VDD) charges: Assessment of the Mulliken, Bader, Hirshfeld, Weinhold, and VDD methods for charge analysis. *J. Comput. Chem.* **25**, 189–210 (2004).

11. Bader, R. F. W. Atoms in molecules. *Acc. Chem. Res.* **18**, 9–15 (1985).

12. Yu, M. & Trinkle, D. R. Accurate and efficient algorithm for Bader charge integration. *J. Chem. Phys.* **134**, 064111 (2011).

13. Tang, W., Sanville, E. & Henkelman, G. A grid-based Bader analysis algorithm without lattice bias. *J. Phys.: Condens. Matter* **21**, 084204 (2009).

14. Slichter, C. P. *Principles of Magnetic Resonance* (Springer Science & Business Media, 2013).

15. Abragam, A. *The Principles of Nuclear Magnetism* (Oxford University Press, 1961).

16. Matsui, H., Mishchenko, A. S. & Hasegawa, T. Distribution of Localized States from Fine Analysis of Electron Spin Resonance Spectra in Organic Transistors. *Phys. Rev. Lett.* **104**, 056602 (2010).
